# A D-lactate dehydrogenase from rice is involved in conferring tolerance to multiple abiotic stresses by maintaining cellular homeostasis

**DOI:** 10.1038/s41598-020-69742-0

**Published:** 2020-07-30

**Authors:** Muskan Jain, Sakshi Aggarwal, Preeti Nagar, Roopam Tiwari, Ananda Mustafiz

**Affiliations:** Laboratory of Plant Molecular Biology, Faculty of Life Sciences and Biotechnology, South Asian University, Akbar Bhawan, Chanakyapuri, New Delhi, 110021 India

**Keywords:** Molecular biology, Plant sciences

## Abstract

D-lactate dehydrogenase (D-LDH) converts D-lactate (the end product of glyoxalase system) to pyruvate and thereby completes the detoxification process of methylglyoxal. D-LDH detoxifies and diverts the stress induced toxic metabolites, MG and D-lactate, towards energy production and thus, protects the cell from their deteriorating effects. In this study, a D-LDH enzyme from rice (OsD-LDH2, encoded by Os07g08950.1) was characterized for its role in abiotic stress tolerance. For this, a combination of in silico, molecular, genetic and biochemical approaches was used. The kinetic analysis revealed OsD-LDH2 to be the most efficient D-LDH enzyme in comparison to D-LDHs from other plant species. Heterologous overexpression of OsD-LDH2 provides tolerance against multiple abiotic stresses in *E. coli*, yeast and plant system. The analysis of D-LDH mutant and OsD-LDH2 overexpressing transgenic plants uncovered the crucial role of D-LDH in mitigation of abiotic stresses. OsD-LDH2 overexpressing plants maintained lower level of ROS and other toxic metabolites along with better functioning of antioxidant system. This is the first report on correlation of D-LDH with multiple abiotic stress tolerance. Overall, OsD-LDH2 emerged as a promising candidate which can open a new direction for engineering stress tolerant crop varieties by maintaining their growth and yield in unfavorable conditions.

## Introduction

Abiotic stress is defined as the impact of non-living factors on the living organisms in a specific environment. Abiotic stress leads to a series of morphological, physiological, biochemical and molecular changes and it has been predicted that environmental factors may limit crop production by approximately 70%^1^. So, to overcome the toxic effects of abiotic stress, a network of events is triggered that starts with stress perception and ends with the expression of a large array of genes. To engineer stress tolerance, various stress responsive genes, proteins and transcription factors have been studied for their role in conferring stress tolerance^2–7^. The present study is aimed at identifying the role of enzyme D-lactate dehydrogenase (D-LDH) in abiotic stress mitigation.

D-lactate dehydrogenase (D-LDH) catalyzes the conversion of D-lactate to pyruvate. D-LDH belongs to the class of D-lactate ferricytochrome c oxidoreductase (EC 1.1.2.4) or D-lactate NAD oxidoreductase (EC 1.1.1.28) depending on the cofactor used, either cytochrome c or NAD. D-LDH enzymes have earlier been identified in prokaryotes and lower fungi where they are involved in anaerobic energy metabolism^8–16^. In *S. cerevisiae* and *K. lactis*, D-lactate cytochrome c oxidoreductase was found important for utilization of D-lactate^13,17,18^. But, extensive information about functioning of D-LDH in higher organisms or plants is yet to be explored.

The major source of D-lactate accumulation in plants is the glyoxalase pathway for methylglyoxal (MG) detoxification. MG is a cytotoxic metabolite produced as a by-product of various metabolic reactions and is accumulated during stress conditions. Accumulated MG reacts with major macromolecules of the cell and disrupts cellular homeostasis^19–22^. To detoxify MG, glyoxalase system consisting of two enzymes, Glyoxalase I and Glyoxalase II converts MG to D-lactate. Another enzyme Glyoxalase III catalyzes the conversion of MG to D-lactate directly^23^. This way MG is detoxified but D-lactate accumulates which is harmful for cellular homeostasis. D-lactate accumulation leads to acidosis causing D-lactate encephalopathy and D-lactate is considered as an indicator of sepsis, trauma and an early marker of intestinal ischemia^24–26^. There is not much information about the same in plants but in a study, the presence of D-lactate in the media was found to negatively affect the seedling development in *Arabidopsis thaliana*^27^.

D-LDH plays an important role in detoxification of D-lactate. D-LDH has already been linked to MG detoxification^17,28–31^**.** The *D-LDH* mutant lines of *Arabidopsis thaliana* were unable to grow in media supplemented with MG indicating role of *D-LDH* in MG metabolization^28^. The in vitro analysis revealed cytochrome c to be a good acceptor of electrons obtained from oxidation of D-lactate^28^. In another study, CYTc was found to be the in vivo acceptor of electrons released from D-lactate oxidation. Overexpression of CYTc and D-LDH was found to increase tolerance to D-lactate and MG^29^. Silencing of *OsD-LDH* in rice resulted in growth inhibition on treatment with MG and also led to the accumulation of MG and D-lactate^30^. The *OsD-LDH* RNAi silenced rice seedlings were salt stress sensitive and showed lower chlorophyll levels in leaves^30^. Recently, we have shown that yeast *D-LDH* mutant is sensitive to MG as well as different abiotic stresses, and Arabidopsis *D-LDH* can complement this mutation. D-LDH has also been shown to be an integral part of the glyoxalase pathway^31^. Thus, D-LDH completes the task of MG detoxification by acting on the end product of glyoxalase pathway. All these studies demonstrate the importance of D-LDH for MG detoxification.

MG level increases by 4–6 fold in response to stress conditions; which is taken care of, by the glyoxalase system^32–36^. MG detoxification would ultimately lead to a proportional rise in the level of D-lactate in the system. Since D-lactate is toxic for the system and D-LDH is the only enzyme capable of neutralizing it, D-LDH might have some role in abiotic stress mitigation. Therefore, an attempt was made to elucidate the role of D-LDH in abiotic stress tolerance.

Using a wide array of in silico, molecular, genetic and biochemical approaches, we have validated the role of rice *D-LDH* in abiotic stress tolerance. The genome wide analysis revealed 3 putative members in the D-LDH family of rice. But, only one of them, OsD-LDH2 was predicted to be active. So, we investigated the active rice D-LDH member for its abiotic stress inducibility. The OsD-LDH2 protein was characterized in detail to find out its substrate specificity and kinetic properties. *OsD-LDH2* could successfully complement yeast *D-LDH* mutant cells. Also, the heterologous overexpression of *OsD-LDH2* provided tolerance against multiple abiotic stresses in *E. coli*, yeast and model plant Arabidopsis. The present study identified *OsD-LDH2* as a major role player in providing multiple abiotic stress tolerance.

## Experimental procedures

### Material and methods

#### In silico identification of D-LDH family in rice

To identify candidate genes encoding D-LDH enzymes, the *Oryza sativa* genome at TIGR, was searched with *Arabidopsis thaliana* D-LDH protein sequence (AtD-LDH encoded by locus At5G06580) with an E value of 1. Since, D-LDH enzyme belong to FAD linked oxidoreductase family of enzymes and require two domains, FAD binding 4 and FAD oxidase c domain, for D-LDH activity; the hits obtained after BLAST were screened one by one and the members belonging to the FAD linked oxidoreductase family of enzymes were selected. These three gene loci were then individually searched, firstly in the locus search function of TIGR, and secondly with BLASTP (https://blast.ncbi.nlm.nih.gov/Blast.cgi), for other information about each one of them.

#### Expression analysis using real time PCR

IR64 seeds were grown hydroponically in growth chamber at 28 ± 2 °C under photoperiod of 16 h and 8 h of dark with 70% humidity. After germination, seeds were grown in Yoshida media for 10 days after which stress were given. For salinity stress, 200 mM of NaCl was added and for drought stress, seedlings were dried with tissue paper for 1 h and 24 h. First strand cDNA was prepared from 2 µg of RNA using Maxima first strand cDNA synthesis kit for qRT-PCR and eIF4α gene used as reference gene. The primers used are listed in Supplementary Table S2. For each sample, three replicates were performed and relative expression ratio was calculated using delta Ct value method.

#### Amplification and cloning of *OsD-LDH2*

RNA was isolated from rice tissue, reverse transcribed to cDNA which was ultimately used for amplifying the two gene fragments using different primer pairs such that there was an overlap of 109 bp between them. Overlapping PCR was used to amplify the full length gene of 1,680 bp (Supplementary Fig. S2). The list of primers used is provided in Supplementary Table S2. The gene was cloned in pJET1.2 cloning vector, pET28a bacterial expression vector and yeast expression vector pYES2. For expression in plant system, full length *OsD-LDH2* gene was cloned in pEARLY100 vector via gateway cloning method.

#### Substrate screening and kinetic characterization

For protein expression, BL21 (pET28a-*OsD-LDH2*) cells were grown at 30 °C till an optical density of 0.5 and then induced with 0.05 mM IPTG for 15 h at 22 °C. The expressed recombinant protein was purified using Ni–NTA affinity chromatography. The substrate specificity was determined using protocol as described earlier^28^. For pH profiling, different buffers (pH 6.5–8.0: MOPS buffer, pH 7.40–9.00: tris buffer, pH 9.00–11.00: sodium carbonate-bicarbonate buffer) were used. Kinetic parameters such as Michaelis–Menten constant (K_m_), catalytic efficiency (K_cat_) and specific activity were calculated. The enzyme activity was measured at different concentrations of D-lactate (5–300 μM) in triplicate.

#### Abiotic stress tolerance assay in *E. coli*

BL21 *E. coli* cells containing the recombinant construct pET28a-*OsD-LDH2* and empty vector (pET28a) were grown in Luria Bertani medium at 37 °C and analyzed for stress tolerance as described previously^37^. As the culture reached optical density of 0.5, the cells were subjected to different stress conditions such as 200 mM NaCl, 5 mM H_2_O_2_, 0.5 mM MG, 100 mM Mannitol and 42 °C heat stress and induced with 0.05 mM IPTG. The growth pattern of cells was noted for 12 h by taking OD at 600 nm after every 2 h time point. The data obtained in triplicates was averaged and used to plot the graph.

#### Functional complementation and stress tolerance in yeast

The *ΔDLD1* mutant BY4741 cells were transformed with expression construct pYES2-*OsD-LDH2* and empty vector pYES2. The transformed cells were streaked (equal volume of secondary cultures with equal OD) on SD Ura^−^ media containing either 20% glucose or 20% galactose with different MG concentrations (0.0–4.0 mM MG) and grown at 30 °C for 72 h.

For checking the stress tolerance of *OsD-LDH2*, the transformed cells were grown in SD Ura^−^ media containing different stressors 1 M NaCl (salinity stress), 25 mM H_2_O_2_ (oxidative stress), 1 mM methylglyoxal (exogenous MG stress), 500 mM mannitol (osmotic stress) and at 42 °C (heat stress) and growth was monitored by taking OD at 600 nm every 5 h interval for 30 h. The entire experiment was replicated and obtained data was averaged to plot the graph.

#### Generation and confirmation of Arabidopsis transgenic plants

*Arabidopsis thaliana* (Col-0 ecotype) were grown in standard growth conditions in growth chamber at 22 °C with photoperiod of 16 h and humidity of 70%. *Arabidopsis thaliana* was transformed with construct pEARLEY100-*OsD-LDH2* via *Agrobacterium* (GV3101) mediated floral dip method^38^. The transgenic plants were confirmed at DNA level by PCR. The seeds of T-DNA insertion mutant for *D-LDH* gene in *Arabidopsis thaliana* (SALK_026859) were procured from TAIR. To confirm the genotype of mutant plants, their genomic DNA was used to set PCR with two set of primers as recommended and shown in Supplementary Fig. S5. The D-LDH activity of total protein extract from mutant, wild type and transgenic plants was checked.

#### Effect of multiple stresses on germination and growth of *D-LDH* mutant and *OsD-LDH2* transgenic plants

The *Arabidopsis thaliana* seeds of mutant (M), wildtype (WT) and *OsD-LDH2* overexpressing transgenic plants (D2TG) were sown on half strength MS agar plates supplemented with different concentrations of various stresses such as D-lactate (0–10 mM), Methylglyoxal (0–1 mM), NaCl (0–150 mM), Mannitol (0–150 mM) and H_2_O_2_ (0–5 mM). These plates were kept at 22 ± 2 °C with photoperiod of 16 h. Monitoring of various growth parameters such as germination rate, survival rate, root length and fresh weight of seedling was followed.

#### Testing transgenic plants for their tolerance against various abiotic stresses

The M, WT and D2TG plants were grown at 22 ± 2 °C with photoperiod of 16 h and humidity of 70% for 25 days and then different stress treatments were given. For 10 days, plants were watered with water for control, 150 mM NaCl for salinity stress, 25 mM H_2_O_2_ for oxidative stress and water was withheld for providing drought stress. The growth parameters including plant height and silique number were measured and tissue was harvested.

#### Measurement of chlorophyll, H_2_O_2_ and O_2_˙¯ levels

For chlorophyll estimation, 100 mg of fresh leaf tissue was taken and ground to powder in liquid nitrogen and then homogenized in 1 ml of DMF (Dimethyl formamide). The extract was centrifuged and supernatant was used to measure optical density at 664 nm and 647 nm. Total chlorophyll, chlorophyll a and chlorophyll b content were calculated using formulas as used earlier^39^.

Detection of H_2_O_2_ was based on histochemical method of DAB (3, 3′- diaminobenzidine) staining. Superoxide radical accumulation in leaf tissue was done by NBT (nitrobluetetrazolium chloride) staining. The results obtained after staining were photographed. The obtained images were subjected to quantitative analysis using ImageJ software. The stain intensity of the leaves (M, WT, D2TG) from different stress conditions was measured and normalized with the stain intensity of the respective control leaves. The obtained values were plotted as relative ROS level.

#### Measurement of methylglyoxal, total glutathione and D-lactate level

Methylglyoxal levels were estimated from 200 mg leaf tissue following the protocol as described earlier^31^. For Glutathione and D-lactate measurement, 200 mg of leaf tissue was taken. The perchloric acid extracted cell lysates were neutralized by 1 M KOH and the neutralized supernatant was used to assay glutathione and D-lactate. For reduced glutathione estimation, 10 μl of neutralized supernatant was added to the reaction mixture containing 140 μl of 0.3 mM NADPH, 20 μl of 6 mM DTNB, 28 μl of 50 mM potassium phosphate buffer pH 7.5 containing 7 mM EDTA, 2 μl of Glutathione reductase (50 U/ml). The reading was taken at 412 nm for 6 min at 2 min interval. To measure the levels of oxidized glutathione, 100 μl of neutralized supernatant was taken in a MCT, to which 2 μl of 2-vinyl pyridine was added and mixed vigorously for 1 min and incubated at 25 °C for 1 h to derivatize reduced GSH to GSSG. Assay to measure the levels of oxidized glutathione was done in similar manner as mentioned above and reading was taken 412 nm. For D-lactate measurement, 50 μl of neutralized extract was added to the reaction mixture containing 100 μl of potassium phosphate buffer, 40 μl of 1 mM DCIP, 10 μl of 60 mM PMS and 1 μl of D-lactate dehydrogenase enzyme (0.025 U/μl). The change in absorbance was recorded at 600 nm for 5 min taking reading at an interval of 30 s. D-lactate content was calculated using a standard curve. For standard curve, different concentrations of D-lactate (0–400 nmoles) were used and assay was performed similarly.

#### Estimation of other abiotic stress related biochemical parameters

200 mg of ground fresh leaf tissue was homogenized in 3 ml of 100 mM phosphate buffer pH 7.8. The homogenate was centrifuged and supernatant was stored as total crude extract at −80 °C. This crude extract was used to estimate various physiological parameters such as MDA, proline and catalase and GST activity^40^.

For malondialdehyde (MDA) estimation, 100 μl of crude extract was added to 1 ml of 0.25% Thiobarbituric acid (TBA) solution and the mixture was boiled in a boiling water bath for 15 min and cooled down on ice for 5 min. Absorbance values were measured at 532 nm and 600 nm. MDA content was calculated from obtained values, given, the extinction coefficient of MDA-TBA at 532 nm is 155. MDA content was expressed as µmoles/ g fresh weight.

To estimate the proline content, 50 μl of crude mix was added to 1 ml of reaction mixture containing 250 μl of 3% sulphosalicylic acid, 250 μl of acetic acid and 500 μl of 2.5% ninhydrin solution. The mixture was boiled in a boiling water bath for 15 min and cooled down on ice for 5 min. Absorbance value was read at 520 nm. The proline content was estimated using a standard curve prepared with varying range (0–100 nmoles) of proline concentration.

Catalase activity was assayed in a reaction mixture containing 155 μl of 30% H_2_O_2_ in 100 ml of 100 mM phosphate buffer pH 7.0. 20 μl of crude extract was added in 200 μl of reaction mixture to initiate the reaction. A decrease in absorbance at 240 nm was monitored for 2 min, taking reading at every 15 s. One unit of catalase is defined as the amount of enzyme that decreases 0.1 of absorbance at 240 nm in 1 min.

For GST activity, the reaction mixture contained 100 μl of 5 mM GSH and 200 μl of 1.5 mM CDNB (1-chloro-2, 4-dinitrobenzene). 30 μl of crude mix was added to 200 μl of reaction mix and the change in absorbance at 340 nm was recorded for 1 min taking reading at every 15 s. One unit of GST is the amount of enzyme that increases the 1 of absorbance at 340 nm in 1 min.

### Statistical analysis

All the data were collected from at least three different experiments of two independent transgenic lines. Data were analyzed by one-way ANOVA and the mean values were compared by Tukey’s test. Statistical analysis was performed using Graphpad prism for windows.

## Results

### In silico analysis identifies multiple D-LDH members in *Oryza sativa*

The BLAST search in the rice genome using Arabidopsis D-LDH (At5g60580), resulted in various hits. When these hits were searched, three members (OsD-LDH1, OsD-LDH2 and OsD-LDH3) were identified in *Oryza sativa* genome. The detail about the size of genes and their co-ordinates are provided in Supplementary Table S1. One of the three members, OsD-LDH3 was found to have three alternative spliced forms. BLASTP search showed only one member (OsD-LDH2) to possess both the domains, FAD binding 4 and FAD oxidase c domains (Supplementary Fig. S1). The putative active protein, OsD-LDH2 encodes for a protein of 560 amino acids with a predicted molecular weight of 62 kDa.

### *OsD-LDH2* is a late inducible gene in response to salinity and drought stress

The relative transcript level of OsD-LDH2 was determined in response to salinity and drought stress in shoot and root tissue of rice seedlings. The real time data shows differential tissue specific stress inducibility of *OsD-LDH2* gene. *OsD-LDH2* gene was downregulated in early time duration of salinity and drought stress in both shoot and root tissues except for the 1 h salinity stress exposed root tissues (Supplementary Fig. S3). However, in late duration stress points, *OsD-LDH2* gene was upregulated in both root and shoot tissues grown in salinity and drought stress conditions (Supplementary Fig. S3).

### OsD-LDH2 preferentially acts on D-lactate with a high substrate affinity

**Figure 1:**
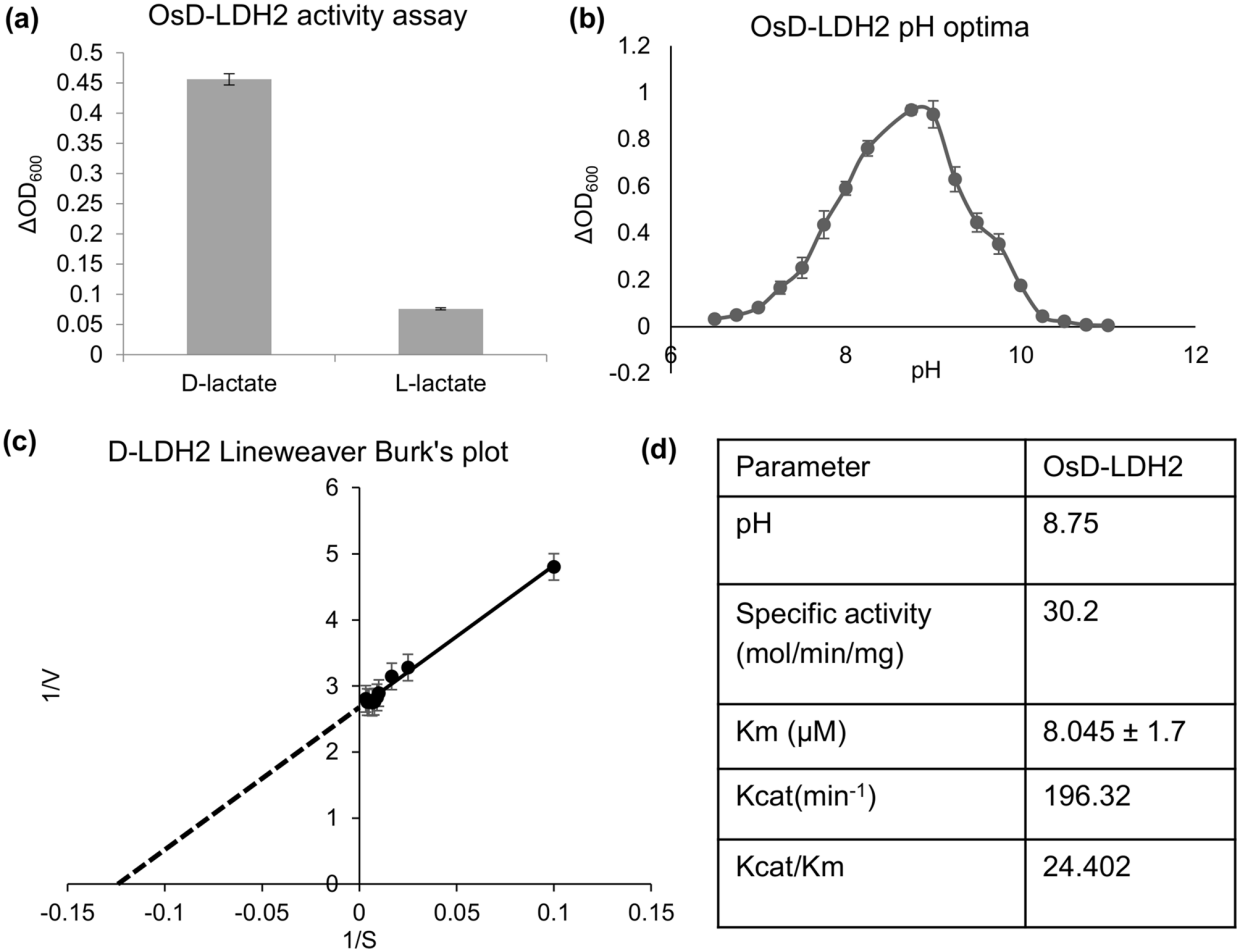
Kinetic characterization of OsD-LDH2 protein: (**a**) Graph depicting the substrate specificity of OsD-LDH2 protein. D-LDH activity assay was checked using E2 fraction with D-lactate and L-lactate as substrate and change in OD was measured at 600 nm. The purified protein showed activity selectively with D-lactate whereas negligible activity was seen with L-lactate. (**b**) Graph showing the effect of pH on the activity of OsD-LDH2. The purified OsD-LDH2 protein was used to check the activity with D-lactate as substrate over a wide range of pH with different buffers (pH 6.5–8.0: MOPS buffer, pH 7.40–9.00: tris buffer, pH 9.00–11.00: sodium carbonate-bicarbonate buffer). OsD-LDH2 showed maximal activity at pH 8.75. (**c**) Lineweaver Burk’s plot depicting the kinetics of OsD-LDH2. D-LDH activity assay was performed using the purified OsD-LDH2 protein with varying concentration of its substrate D-lactate. The obtained data was used to plot Lineweaver Burk’s plot and various kinetic parameters were calculated. (**d**) Table showing the various kinetic parameters calculated for OsD-LDH2 protein.

The OsD-LDH2 protein was expressed in BL21 cells and purified via affinity chromatography. The purified protein gave a band around 62 kDa on SDS gel. To identify the substrate for OsD-LDH2, the purified protein was used to check activity with D-lactate and L-lactate as substrates. OsD-LDH2 showed quite high activity with D-lactate and negligible activity was seen with L-lactate (Fig. 1a). To identify the optimum pH for OsD-LDH2, its activity was checked in different buffers with a wide range of pH. The optimal pH for OsD-LDH2 activity was found to be 8.75 (Fig. 1b). The kinetic profile of the enzyme was measured and Lineweaver Burk’s plot was made (Fig. 1c). Various kinetic parameters such as K_m_, K_cat_, K_cat_/K_m_ were calculated to analyze the kinetic potential of the enzyme (Fig. 1d). The specific activity of OsD-LDH2 was calculated to be 30.2 mol/min/mg of protein.

### Overexpression of *OsD-LDH2* confers multiple stress tolerance to *E. coli*

**Figure 2:**
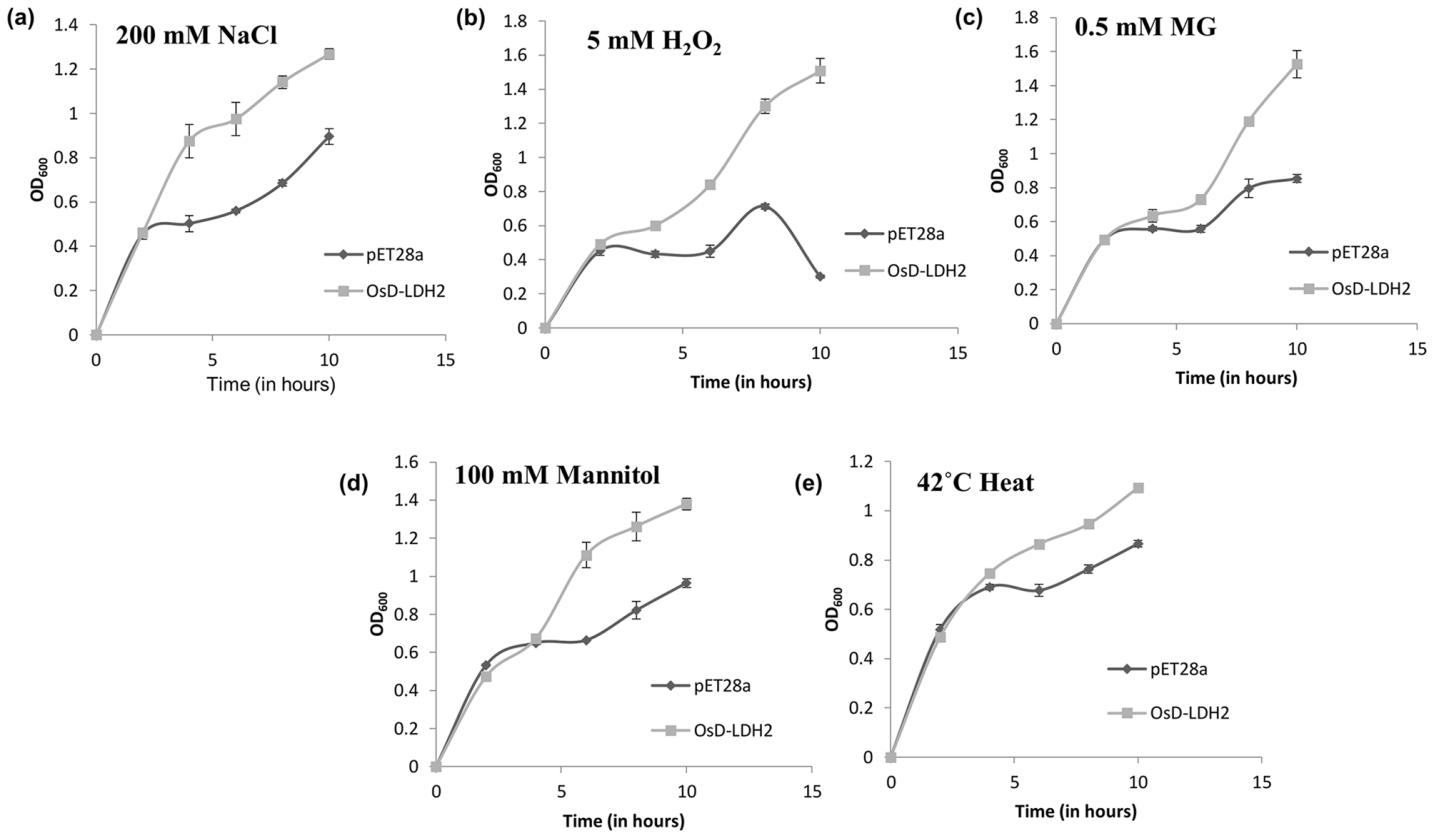
Stress tolerance assay of *E. coli* cells overexpressing OsD-LDH2 protein: The *E. coli* cells transformed with *OsD-LDH2* were grown in presence of (**a**) Salinity stress (200 mM NaCl), (**b**) Oxidative stress (5 mM H_2_O_2_), (**c**) Cytotoxic Methylglyoxal stress (0.5 mM MG), (**d**) Osmotic stress (100 mM Mannitol) and (**e**) Heat stress (42 °C) and their growth was monitored over time. Cells transformed with empty vector (pET28a without any gene) were used as control.

The link between *OsD-LDH2* and stress tolerance was primarily assessed in a unicellular prokaryotic system, *E. coli*. The OsD-LDH2 expressing BL21 cells were grown in presence of various abiotic stresses such as salinity, oxidative, osmotic conditions and their growth pattern was monitored in comparison to BL21 cells containing empty vector pET28a. The cells overexpressing *OsD-LDH2* gene were tolerant to the presence of various stresses as they grew better than the control cells (Fig. 2). Thus, *OsD-LDH2* gene provided tolerance to multiple abiotic stresses.

### *OsD-LDH2* functionally complements and provides multiple stress tolerance to yeast *DLD1* mutant (*ΔDLD1*) cells

**Figure 3:**
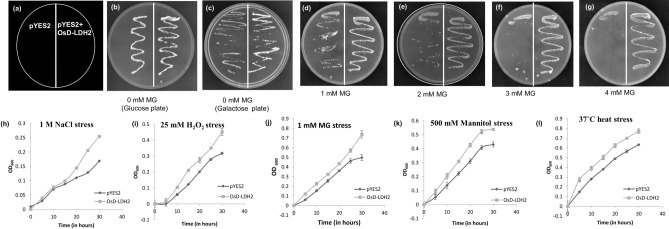
Functional complementation and stress tolerance assay using the yeast *ΔDLD* mutant cells: The *Saccharomyces cerevisiae D-LDH* mutant cells were transformed with constructs pYES2-*OsD-LDH2* and empty vector pYES2. The transformed cells were grown on solid Ura- SD media with various supplements to check for functional complementation of mutant yeast cells transformed with *OsD-LDH2* gene. (**a**) Pictorial depiction of various strains used. (**b**) Medium supplemented with 20% glucose; (**c**) Medium supplemented with 20% galactose; (**d**) Medium supplemented with 20% galactose and 1 mM MG; (**e**) Medium supplemented with 20% galactose and 2 mM MG; (**f**) Medium supplemented with 20% galactose and 3 mM MG; (**g**) Medium supplemented with 20% galactose and 4 mM MG. The transformed cells were grown in liquid Ura- SD media supplemented with 20% galactose in the presence of (**h**) Salinity stress (1 M NaCl), (**i**) Oxidative stress (25 mM H_2_O_2_), (**j**) Cytotoxic Methylglyoxal stress (1 mM MG), (**k**) Osmotic stress (500 mM Mannitol) and (**l**) Heat stress (37 °C). The growth pattern was monitored spectrophotometrically for 30 h. Cells transformed with empty vector (pYES2 without any gene) were used as control.

Since, *DLD1* mutant yeast cells are methylglyoxal sensitive, mutant cells transformed with pYES2-*OsD-LDH2* construct and empty vector pYES2 were grown in media supplemented with MG to assess the ability of *OsD-LDH2* to complement *DLD1* mutation in yeast. In the absence of MG, growth was seen in both the transformants in the media containing either glucose or galactose (Fig. 3). The *DLD1* mutant yeast cells containing empty vector grew till 1 mM MG (Fig. 3d) whereas the cells containing *OsD-LDH2* gene tolerated quite higher MG concentrations, upto 4 mM (Fig. 3g).

To assess the stress tolerance ability of yeast cells upon heterologous expression of *OsD-LDH2*, the *DLD1* mutant cells transformed with empty vector pYES2 and pYES2-*OsD-LDH2* were grown in liquid Ura^−^ media supplemented with different abiotic stress conditions (salinity, oxidative, exogenous MG, osmotic and heat). In all the stress conditions under study, the cells overexpressing *OsD-LDH2* gene showed more tolerance in comparison to the cells with empty vector (Fig. 3h–l). Thus, overexpression of OsD-LDH2 can provide tolerance to multiple abiotic stresses in yeast too.

### *OsD-LDH2* overexpressing Arabidopsis transgenic plants show enhanced detoxification of D-lactate and MG

The putative transgenic lines obtained were confirmed at both DNA and enzyme level (Supplementary Fig. S4). The *D-LDH* mutant T-DNA insertion Arabidopsis line (SALK_026859) procured from TAIR was confirmed by PCR and activity assay (Supplementary Fig. S5). The transgenic plants showed almost 40% more D-LDH activity in comparison to the wild type plants and the mutant plants showed ~ 60% less D-LDH activity than the wild type Arabidopsis plants (Supplementary Fig. S4).

**Figure 4:**
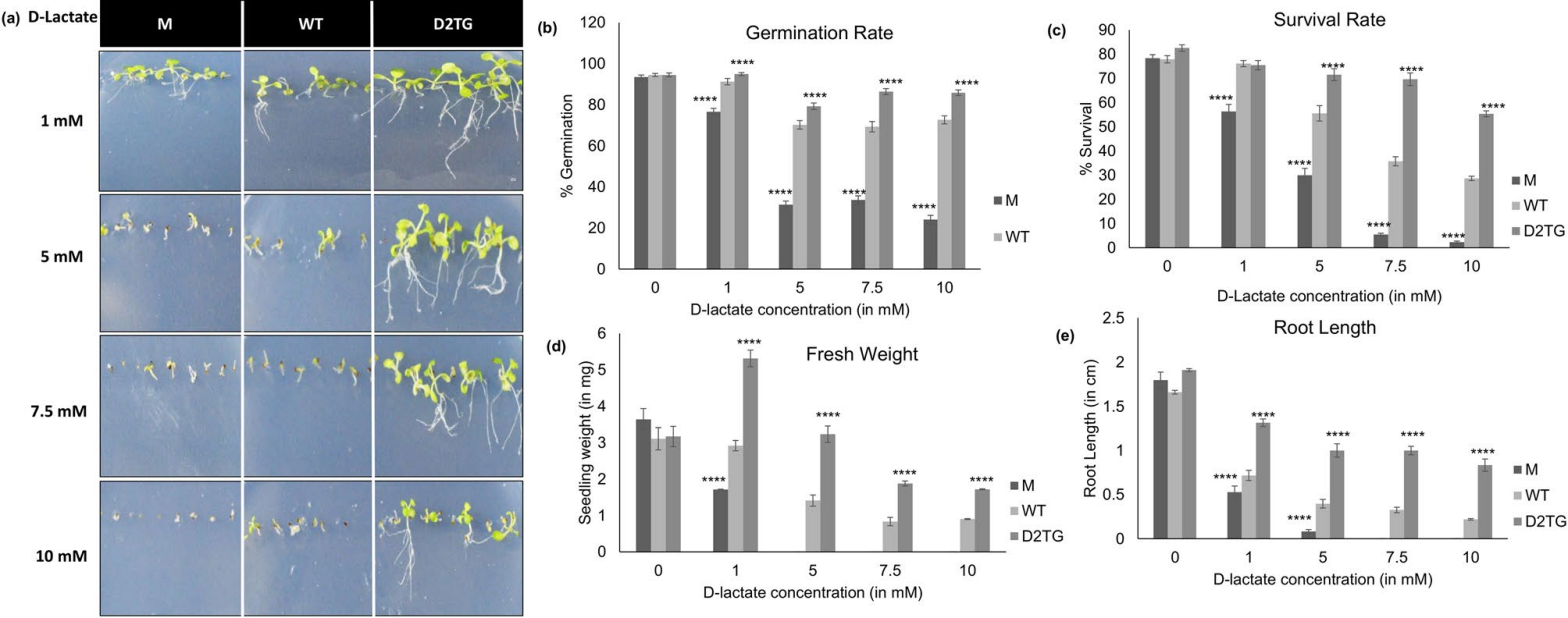
Effect of exogenous D-lactate on *D-LDH* mutant and *OsD-LDH2* overexpressing transgenic plants. The *D-LDH* mutant, wild type and *OsD-LDH2* overexpressing Arabidopsis transgenic seeds were sown in half strength MS media supplemented with different concentrations of D-Lactate and their growth was monitored. (**a**) 2-week old *D-LDH* mutant and transgenic plants grown in varying concentrations of D-lactate. M: *D-LDH* mutant, WT: wild type, D2TG: *OsD-LDH2* transgenic line. Graphs depicting various growth parameters such as (**b**) germination rate, (**c**) survival rate, (**d**) fresh weight and (**e**) root length were calculated. Each value represents mean ± SD of at least three different experiments. Statistically significant differences were determined using one-way ANOVA as compared with wild type plants under similar conditions and indicated by ****P < 0.0001, ***P < 0.001, **P < 0.01, *P < 0.05.

The homozygous *D-LDH* mutant plants (M), wild type Arabidopsis plants (WT) and the *OsD-LDH2* overexpressing single insertion homozygous transgenic Arabidopsis plants (D2TG) were analyzed for their D-lactate and MG detoxification capabilities. The M, WT and D2TG seeds were sown on solid MS media supplemented with different concentrations of D-lactate and MG. In control conditions, MS media without D-lactate or MG, no evident difference in germination and growth rate of M, WT or D2TG was observed. But, there was striking phenotypic difference in presence of MG or D-lactate. With the increasing concentrations of D-lactate (1–10 mM), the germination rate and survival rate of mutant seedlings decreased by ~ 75% and wild type seedlings showed ~ 50% decrease in their survival rate whereas the transgenic seedlings maintained their growth similar to control conditions even at high D-lactate concentration of 10 mM (Fig. 4b,c). The mutant and wild type seeds only germinated and could not grow at higher (5–10 mM) D-lactate concentrations, consequently, their fresh weight and root length showed similar pattern (Fig. 4d,e).

**Figure 5:**
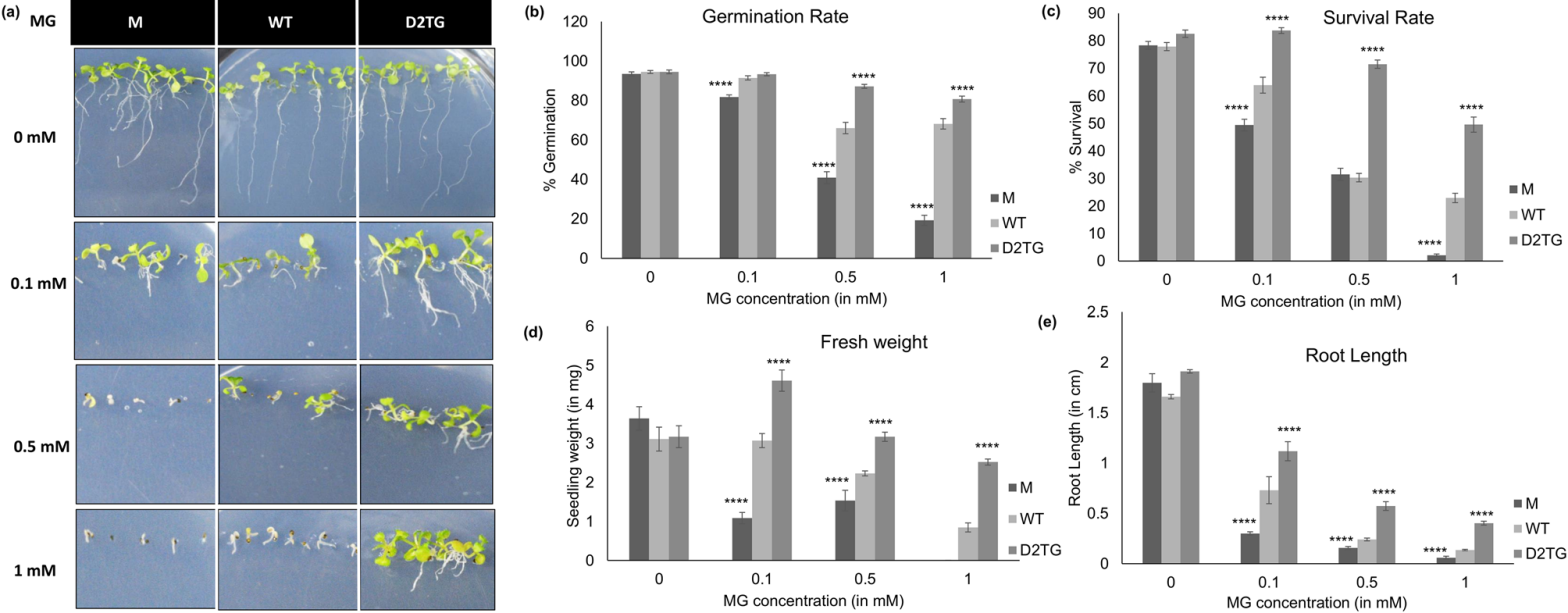
Effect of exogenous methylglyoxal (MG) on *D-LDH* mutant and *OsD-LDH2* overexpressing transgenic plants. The *D-LDH* mutant, wild type and *OsD-LDH2* overexpressing Arabidopsis transgenic seeds were sown in half strength MS media supplemented with different concentrations of MG and their growth was monitored. (**a**) 2-week old *D-LDH* mutant and transgenic plants grown in varying concentrations of MG. M: *D-LDH* mutant, WT: wild type, D2TG: *OsD-LDH2* transgenic line. Graphs depicting various growth parameters such as (**b**) germination rate, (**c**) survival rate, (**d**) fresh weight and (**e**) root length were calculated. Each value represents mean ± SD of at least three different experiments. Statistically significant differences were determined using one-way ANOVA as compared with wild type plants under similar conditions and indicated by ****P < 0.0001, ***P < 0.001, **P < 0.01, *P < 0.05.

**Figure 6:**
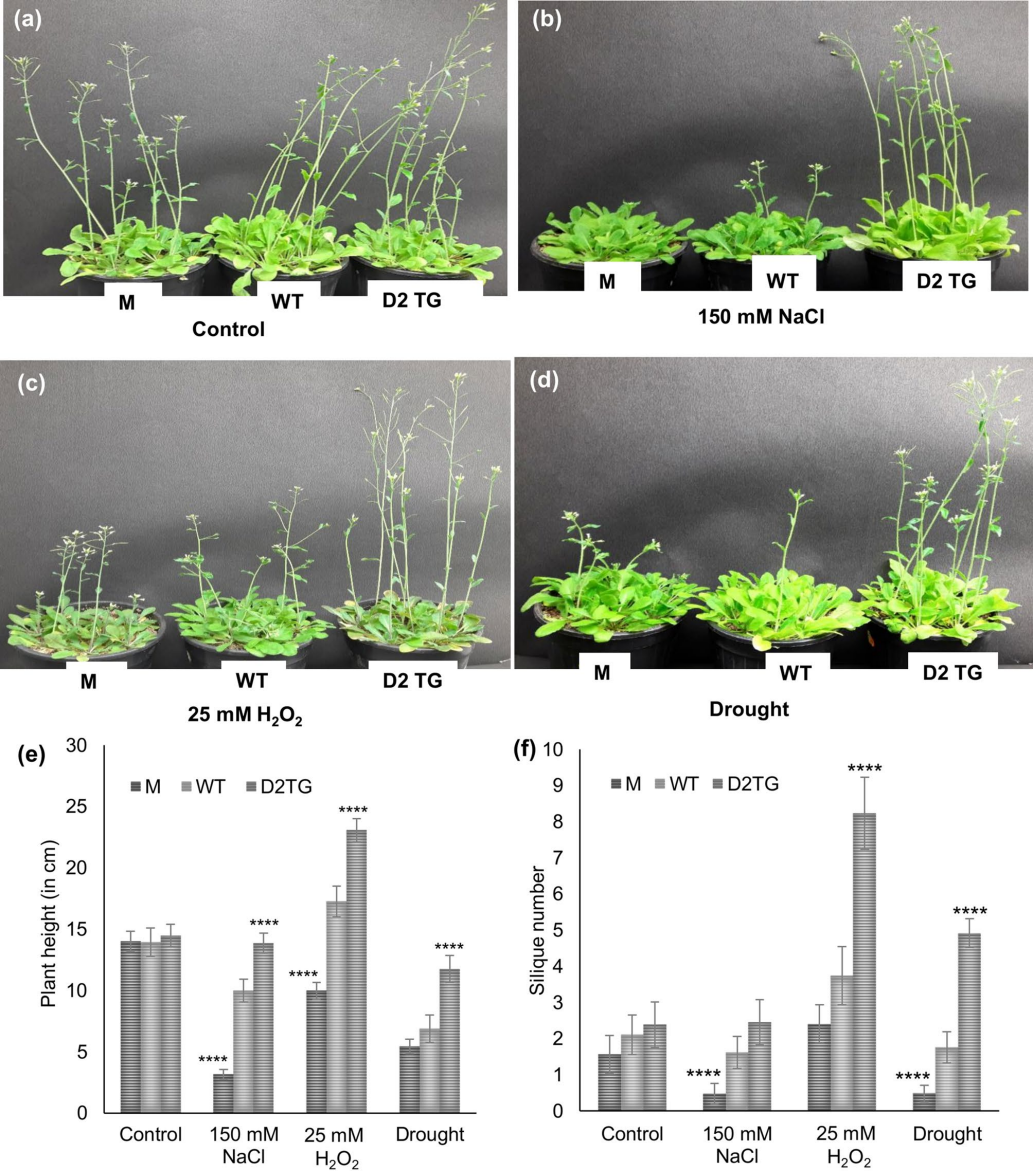
Growth profile and yield parameters of *D-LDH* mutant and *OsD-LDH2* overexpressing transgenic plants in response to various abiotic stresses. The *D-LDH* mutant, wild type and *OsD-LDH2* overexpressing Arabidopsis transgenic plants were grown in presence of various abiotic stress conditions (salinity, oxidative and drought) along with the wild type Arabidopsis plants. (**a**) For control, plants were given water only, whereas, (**b**) salinity stress was given by watering the plants with 150 mM NaCl, (**c**) 25 mM H_2_O_2_ for oxidative stress and (**d**) watering was withheld for providing drought stress. Growth and yield parameters such as (**e**) plant height, (**f**) silique number were measured from the corresponding plants and represented as mean ± SD of at least three different experiments. Statistically significant differences were determined using one-way ANOVA as compared with wild type plants under similar conditions and indicated by ****P < 0.0001, ***P < 0.001, **P < 0.01, *P < 0.05.

Similar to D-lactate, MG showed dose dependent toxicity to the mutant seedlings but mildly effected the D2TG plants (Fig. 5a). The D2TG seeds germinated and could grow till 1 mM of MG concentration and their germination and survival rate was almost similar to control conditions (Fig. 5b,c). At higher concentrations of MG (1 mM), the fresh weight and root length of the transgenic plants was much higher in comparison to the mutants and the wildtype seedlings (Figs. 5d and 6e). The mutant seedlings showed ~ 70% decrease in fresh weight and ~ 83% decrease in the root length with increasing MG concentrations.

### *OsD-LDH2* transgenic plants perform better than *D-LDH* mutant and wildtype plants in multiple abiotic stresses

The 25 days old M, WT and D2TG plants were subjected to various abiotic stresses (Fig. 6a–d). Their phenotypic parameters were measured and harvested shoot tissue was subjected to estimation of various physiological and biochemical parameters.

In control condition, growth of M, WT and D2TG plants was comparable with almost similar height and silique number. In all the stresses, there was a significant increase in the height of D2TG plants and decrease in the height of M plants, in comparison to the WT plants (Fig. 6e). The height of D2TG plants in oxidative stress was ~ 66% more than even the control plants. Silique number followed a similar pattern as of plant height in all the stress conditions under study (Fig. 6f). Again, the number of silique in D2TG plants grown in oxidative stress was almost two times higher than any of the plants.

### OsD-LDH2 overexpression lowers the accumulation of ROS and other toxic metabolites in plants

**Figure 7:**
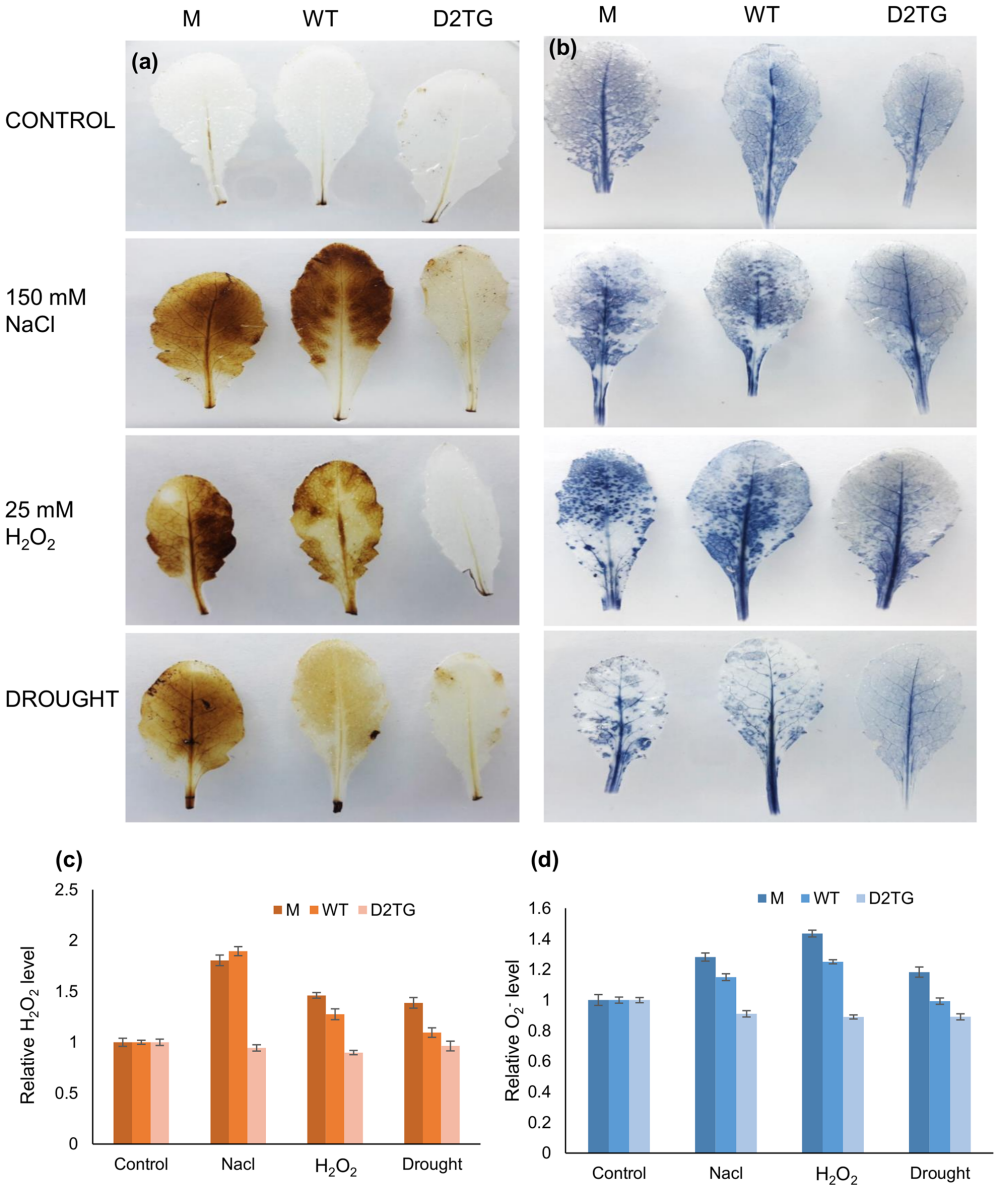
In situ detection of H_2_O_2_ and O_2_˙¯ production in the *D-LDH* mutant and *OsD-LDH2* overexpressing transgenic plants in response to multiple abiotic stresses. The *D-LDH* mutant, wild type and *OsD-LDH2* overexpressing Arabidopsis transgenic plants were grown in presence of various abiotic stress conditions (salinity, oxidative and drought). The leaf tissue from the mutant, wild type and transgenic plants was used to visualize (**a**) H_2_O_2_ and (**b**) O_2_˙¯ using DAB and NBT staining. The stain intensity of leaves from different stress conditions was quantified and normalized using the level of control leaves. The (**c**) relative H_2_O_2_ content and (**d**) relative O_2_^.-^ level clearly indicate the presence of lower ROS levels in the transgenic plants.

All the stress conditions are known to increase ROS production. H_2_O_2_ and O_2_˙¯ are the primary ROS produced in response to stresses. The H_2_O_2_ levels were detected by staining leaves with DAB. In control conditions, all the leaves from M, WT and D2TG were visibly clear with little stain. In the stress conditions, the M and WT leaves showed intense brown staining signifying increased H_2_O_2_ accumulation whereas the D2TG leaves were almost clear (Fig. 7a). The level of superoxide ions (O_2_˙¯) was determined by NBT staining. In control conditions, all the leaves appeared almost similar with very light stained intensity. However, in the stress conditions, the M and WT leaves showed blue staining spots while the D2TG leaves were less stained. This shows more superoxide ions accumulation in mutant leaves (Fig. 7b). Similar pattern is observed quantitatively with the transgenic leaves showing lower ROS accumulation in presence of different abiotic stresses (Fig. 7c,d).

**Figure 8:**
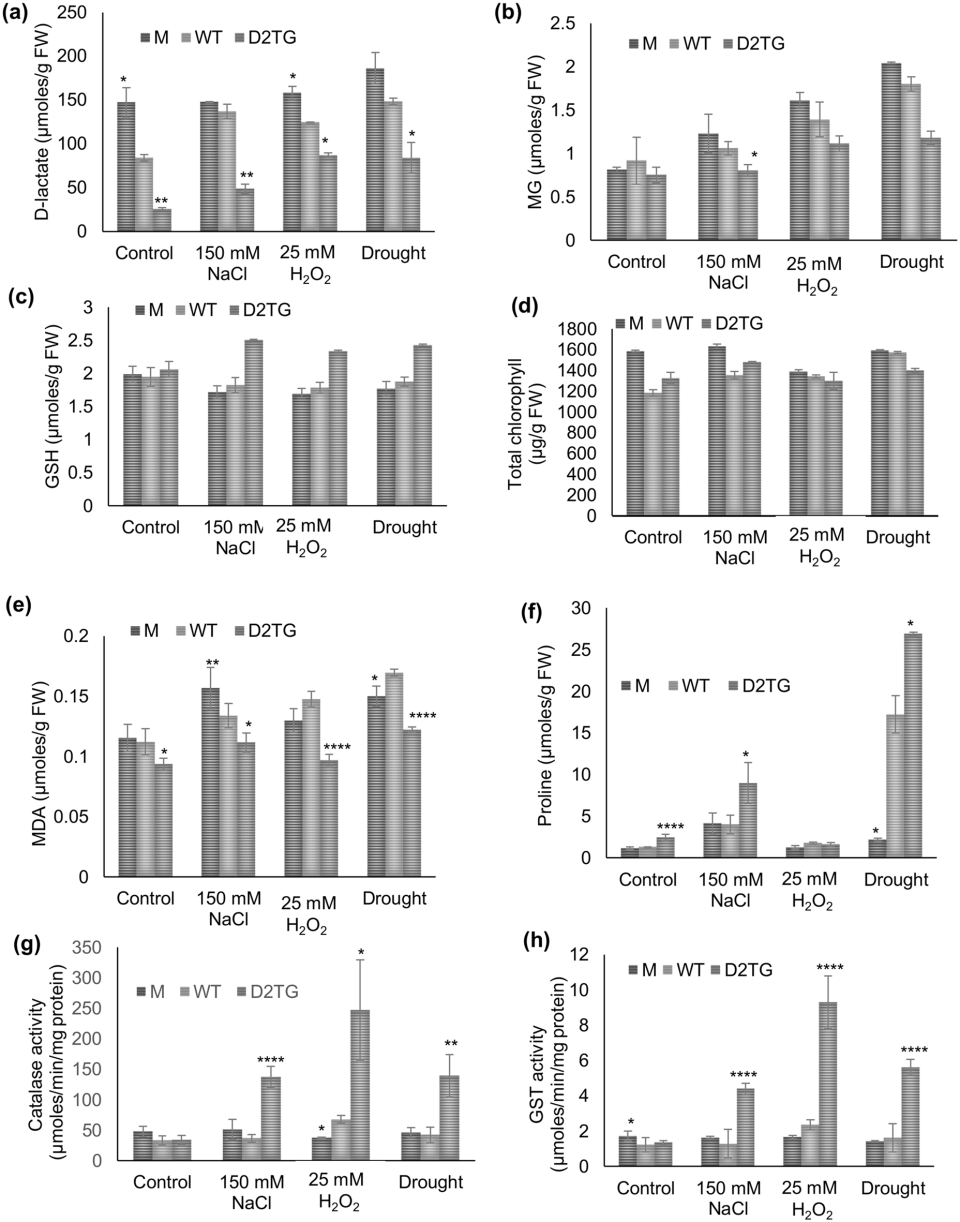
Comparison of various physiological parameters of the *D-LDH* mutant and *OsD-LDH2* overexpressing transgenic plants in response to multiple abiotic stresses. The *D-LDH* mutant, wild type and *OsD-LDH2* overexpressing Arabidopsis transgenic plants were grown in presence of various abiotic stress conditions (salinity, oxidative and drought). The leaf tissue from the mutant, wild type and transgenic plants was used to estimate (**a**) D-lactate content (**b**) methylglyoxal level, (**c**) total glutathione content, (**d**) total chlorophyll content, (**e**) MDA content, (**f**) proline content, (**g**) Catalase activity and (**h**) GST activity. Each value represents mean ± SD of at least three different experiments. Statistically significant differences were determined using One-way ANOVA as compared with wild type plants under similar conditions and indicated by ****P < 0.0001, ***P < 0.001, **P < 0.01, *P < 0.05.

As the direct substrate for D-LDH is D-lactate, the levels of D-lactate in various stress conditions were estimated in M, WT and D2TG plant tissue. The D-lactate levels were 56% more in the mutant tissue and 74% lower in the transgenic tissue in comparison to the WT even in the control conditions (Fig. 8a). The salinity, oxidative and drought stress led to 63%, 48% and 76% increase in D-lactate level in WT plants respectively in comparison to the control value but D2TG plants maintained lower D-lactate levels as in control condition. In comparison to the D-lactate level of WT plants, M plants had significantly higher level (8% in salinity, 27% in oxidative and 25% more in drought stress) and the D2TG plants have significantly lower level (64% in salinity, 29% in oxidative and 43% in drought stress) (Fig. 8a).

Since, D-lactate is formed as a result of MG detoxification pathway, MG levels were measured. In the control condition, MG content was almost similar in M, WT and D2TG plants. But in all the stress conditions, MG level was higher in mutant plants in comparison to the wild type plants of the same stress (Fig. 8b). However, the D2TG plants successfully maintained quite lower level of MG in different abiotic stress conditions studied (Fig. 8b).

### OsD-LDH2 enables antioxidant system to function properly in presence of multiple abiotic stress conditions

To gain insight into the cellular redox status, total glutathione levels were estimated. In control condition, the total glutathione levels were similar in M, WT and D2TG plants. The total glutathione content was lower in M plants but higher in D2TG plants as compared to WT plants of any stress condition (Fig. 8c). The transgenic plants maintained better redox pool than the M and WT plants in various abiotic stress conditions.

The estimation of total chlorophyll content revealed almost similar chlorophyll content in D2TG, M and WT plants in all conditions (Fig. 8d).

MDA is produced by decomposition of lipid peroxides and is referred to as the marker of oxidative lipid injury. In control condition, the MDA content was similar in M and WT but the D2TG plants showed significantly lower MDA levels. In all the stress conditions studied, MDA levels were higher in M and WT plants in comparison to the control conditions. But, the D2TG plants maintained a lower MDA level in all the stress conditions implying a lower membrane damage in the transgenic plants (Fig. 8e).

Proline acts as an osmoprotectant and antioxidant in times of stress. In salinity and drought stress, proline level increased in all M, WT and D2TG plants in comparison to their level in control condition. But among these three, D2TG plants showed an extremely high level (2 and 1.5 folds increase respectively) of proline content in salinity and drought stress (Fig. 8f). However, in oxidative stress the proline levels were not much affected.

All the stresses are associated with increased ROS production and the induction of antioxidant enzymes and system is the only strategy to save from harmful effects of ROS. Therefore, catalase and GST activity were also measured. In control conditions, catalase and GST activity were in the same range in M, WT and D2TG plants. Stress conditions led to significant increase in the activity of both the antioxidant enzymes particularly in response to oxidative stress. The catalase activity of D2TG plants increased by 3.8, 3.6 and 3.3 folds in salinity, oxidative and drought stresses respectively (Fig. 8g). The GST activity increased by 3.5, 4 and 3.5 folds in D2TG plants in response to salinity, oxidative and drought stresses respectively (Fig. 8h). The M and WT plants had quite low levels of catalase and GST activity. The transgenic plants showed comparatively higher induction of antioxidant enzymes.

## Discussion

In the present study, an attempt was made to explore the role of rice D-lactate dehydrogenase (D-LDH) in multiple abiotic stress mitigation. The rice genome contains multiple D-LDH encoding genes (Supplementary Table S1). The alternative spliced forms were found for OsD-LDH3 only (Supplementary Table S1). However, a previous study indicates the presence of an alternative spliced form for OsD-LDH1 also^30^. The bioinformatic analysis revealed only OsD-LDH2 to be active and was studied in detail to determine its role in stress tolerance. *OsD-LDH2* was found to be stress inducible. Its expression level increased with prolonged duration of salinity and drought stresses (Supplementary Fig. S3). This was an indication that in response to salinity and drought stresses, D-LDH might play a crucial role.

D-LDH enzymes are supposed to act specifically on the D-isomer of lactate and not on L-lactate. L-lactate is oxidized by L-LDH (L-lactate dehydrogenase). L-LDH and D-LDH belong to evolutionarily unrelated enzyme families^41,42^. The L-LDH class of enzymes has been extensively studied but much is not known about the D-LDH enzyme family^43,44^. OsD-LDH2 showed clear preference for D-lactate in comparison to the L-isomer (Fig. 1). This predisposition of OsD-LDH2 for D-lactate over L-lactate resembles those of other known D-LDHs belonging to *A. thaliana, S. cerevisiae, R. palustris, and P. elsdenii*^9,16,28,45^. The kinetic analysis revealed OsD-LDH2 has the lowest k_m_ (~ 8 μM) and highest k_cat_/k_m_ value (24.402 × 10^6^) among the other known D-LDHs from the plant system (Table 1). This demonstrates OsD-LDH2 to be the most active D-LDH enzyme characterized from plants. *OsD-LDH2* was successful in complementing the yeast *DLD1* mutant (Fig. 3a–g). The yeast D-LDH is targeted to mitochondria^13^. In silico analysis shows the presence of a N-terminal mitochondrial localization tag in OsD-LDH2. OsDLDH2 was able to complement the MG sensitive phenotype of yeast *DLD1* mutant cells rendering them MG tolerant upon complementation (Fig. 3). This indicates that OsD-LDH2 is also localized to mitochondria, thereby complementing the yeast D-LDH function enabling yeast cells to become MG tolerant. On heterologous expression in *E. coli*, yeast and Arabidopsis, *OsD-LDH2* provided tolerance to multiple abiotic stresses (Figs. 2, 3 and 6). In a previous study, the *D-LDH* Arabidopsis mutant has been found to be sensitive to the presence of MG and D-lactate in the media^28^. In the present study, we have compared the effects of OsD-LDH2 overexpression with the *D-LDH* mutant and wildtype plants. The presence of MG and D-lactate in the media seemed to affect the germination and growth rate of M and WT seedlings drastically, whereas the D2TG seedlings maintained their growth (Figs. 4 and 5).

**Table 1:** A comparison of the kinetic parameters of OsD-LDH2 with other known D-LDH enzymes from plants.

Parameter	OsD-LDH2 (Os07g08950.1)	OsD-LDH (Os07g06890)	AtD-LDH (At5g06580)
Km (µM) (with DCIP as co-substrate)	8.045 ± 1.7	68.19 ± 5.38	317 ± 31
Kcat (min^−1^)	196.32	156	73
Kcat/Km (min^−1^/M^−1^)	24.402 × 10^6^	2.28 × 10^6^	0.231 × 10^6^
References	This study	^30^	^28^

All the abiotic stresses cause a disruption of cellular homeostasis ultimately leading to a decrease in growth and productivity of plants. The D2TG plants possessed more height and silique number than the WT and M plants when grown in presence of salinity, oxidative and drought stress conditions (Fig. 6e,f). The D2TG plants exhibited better phenotype in oxidative stress, being taller and having much higher number of siliques than WT and mutant plants. This might be due to the antioxidant properties of pyruvate, the product of D-LDH. Pyruvate embodies antioxidant properties due to its α-keto carboxylate structure which enables it to directly and non-enzymatically neutralize peroxides and peroxynitrites. H_2_O_2_ is the major source of oxidative stress. Pyruvate forms acetate, carbon-dioxide and water by reacting with H_2_O_2_^46^. Thus, pyruvate directly detoxifies H_2_O_2_ leading to better growth of the plants. Previously, the detached leaves of RNAi silenced *OsD-LDH* rice seedlings showed sensitive phenotype when floated onto 200 mM NaCl. These leaves also showed drastically decreased levels of chlorophyll in comparison to the wild type leaves^30^. A similar effect is seen on the *D-LDH* mutant Arabidopsis plants in the present study. The mutant plants show diminished growth in presence of different abiotic stresses. The primary effects of any stress condition are displayed on the photosynthetic machinery and phenotype of plants^47^. Here, the *D-LDH* mutant plants showed smaller leaves as a coping mechanism, and also flowered early on exposure to stress conditions. In times of stress, plants reduce the leaf size as smaller leaf area transpires less water. This is a mechanism to survive in stress conditions by maintaining the limited water supply in soil for longer periods^48^.

Abiotic stresses are inevitably associated with increased ROS production and accumulation. H_2_O_2_ is the first stable compound produced in the cells as a result of stress^49^. The levels of major ROS, hydrogen peroxide (H_2_O_2_) and superoxide radical (O_2_˙¯) were detected by staining them using specific dyes. The mutant and wildtype leaves showed more ROS accumulation in all the stress conditions (Fig. 7). ROS induce cellular damage by degradation of proteins, inactivation of enzymes, alterations in gene expression and interfering in various pathways of metabolic importance^50^. As a general adaptation strategy, plants use induction of different antioxidant enzymes to overcome stresses^49^. A concerted action of both enzymatic (catalase, SOD, POD etc.) and non-enzymatic (Glutathione, MDA etc.) antioxidant defense system works against the stressors. SOD converts superoxide to H_2_O_2_ whereas the detoxification of H_2_O_2_ is carried out by APX, GPX and catalase^51^. The D2TG plants showed extremely higher catalase and GST activity as compared to M and WT plants in different abiotic stress conditions (Fig. 8g,h). GSH is a well-known antioxidant and the potential scavenger of toxic ROS like H_2_O_2_ and superoxide radicals^52–54^. Also, GSH is a major cofactor in detoxification of MG. The D2TG plants maintained a higher level of total GSH (Fig. 8c). However, significant changes were not observed in case of GSH/GSSG ratio (data not shown). Here, *D-LDH* gene was overexpressed and in this pathway, the levels of GSH are affected by GLY enzymes. GLYI utilizes GSH for the first reaction and that GSH is recycled back by the GLYII enzyme. Probably because of this, significant change in the GSH/GSSG ratio was not observed. MDA reflects the level of lipid peroxidation by ROS and indicates the prevalence of free radical reaction in the tissues. MDA relates inversely to membrane stability^55,56^. The D2TG plants maintained a lower MDA level in different stress conditions whereas comparatively more lipid peroxidation and membrane damage was observed in M and WT plants (Fig. 8e). Increased amount of organic solutes is important for osmotic adjustment. Proline acts as an osmolyte, metal chelator, contributes to stabilization of proteins, membranes, subcellular structures, scavenging free radicals, buffering cellular redox potential^57–59^. Also, proline modulates gene expression and cellular functions, thus, acting as stress related signal in a variety of stress conditions. D2TG plants exhibited higher proline content than the M and WT plants in salinity and drought stress conditions. This might be due to the complex metabolic link between proline and pyruvate, the product of D-LDH. This pyruvate serves multiple functions inside the cell. One of them is leading to an increase in proline concentrations. Pyruvate goes to TCA cycle, from where α-ketoglutarate is produced, which is then, converted to glutamine and ultimately leads to formation of proline^59,60^. In this way, a direct link between pyruvate and proline exists. Although this link has not been explored in detail yet, but this can be the reason for increase in proline level in D2TG plants. Earlier studies have also indicated the overexpression of various enzymes, transcription factors as well as ion pumps led to rise in proline levels contributing to better stress tolerance^4,61–64^. But, in oxidative stress, proline levels were largely unaffected (Fig. 8f). This probably has arisen because of prevalence of other detoxification mechanisms in case of oxidative stress as D-LDH possibly has some special role in oxidative stress tolerance.

MG levels are known to increase by 4–6 folds in response to various stress conditions^32,33,36^. In the present study too, upto 2 to 2.5-fold increase in MG levels was observed in M and WT plants in response to different abiotic stresses. But the D2TG transgenic plants maintained lower MG levels. This is attributed to non-availability of D-lactate for end product inhibition on the activity of GLYII. In a previous study, D-lactate and GSH were found to exhibit end product inhibition on the activity of GLYII^65^. In another study, in the RNAi silenced rice seedlings sown on MS media with MG, decreased activity of GLYI was found^30^. The reaction of GLYI is regarded as the rate limiting step for MG detoxification. Consequently, decreased GLYI activity was found consistent with increased MG content. This indicates that knockdown of D-LDH changes the behavior of GLYI. In our case, overexpression of D-LDH is driving the reaction forward leading to faster detoxification of MG. MG is converted to D-lactate by GLYI and GLYII, but the process of MG detoxification is completed by D-LDH. When abundant amount of D-LDH is present, it catalyzes D-lactate converting it to pyruvate and diverts it to TCA cycle. Thus, D-lactate is unable to inhibit GLYII and the glyoxalase cycle moves faster and detoxifies more of MG. In this way, due to increased detoxification, there is lower MG level in the transformed plants.

The effect of abiotic stress on D-lactate levels is largely unknown. Here, for the first time, we have reported increase in levels of D-lactate in response to various abiotic stresses (Fig. 8a). In WT plant tissue, there was 63%, 48% and 76% increase in the D-lactate level on exposure to salinity, oxidative and drought stresses respectively. The transgenic plants have however, maintained quite lower level of D-lactate. D-lactate level was observed to increase in *D-LDH* silenced rice seedlings on exposure to MG^30^. D-lactate that accumulates in the stress conditions, is toxic for the system. D-lactate toxicity has not been reported in plants but there are a few studies in animals and humans. Accumulation of D-lactate generates acidic pH and causes D-lactate acidosis or D-lactate encephalopathy. D-lactic acidosis in blood can cause neurologic symptoms such as delirium, ataxia and slurred speech^26^. D-lactic acidemia has been linked with grain overload in ruminants, short bowel syndrome in humans and diarrhea in calves. Subclinical elevation of D-lactate is regarded as an indicator of sepsis, trauma^24^ and an early marker of intestinal ischemia^25^. In plants, D-lactate has been found to effect the growth of *Arabidopsis thaliana* negatively in a concentration dependent manner, arresting the growth of seedling shortly after germination^27^. Similar effects were observed in this study. The *D-LDH* mutant plants had higher levels of D-lactate and their growth was diminished. The mutant plants could not grow or even germinate in many stress conditions, because of the poor health status of plants owing to higher D-lactate accumulation and toxicity. So, D-lactate accumulates in stress conditions and is toxic for the cells. The detoxification of D-lactate is a major requirement for plants to grow in times of stress and that is the function of D-LDH enzyme. The plants overexpressing D-LDH grew comparatively better and also maintained a better antioxidant pool along with lesser toxic metabolites (Fig. 8).

The D2TG plants grew better in presence of stress in comparison to the absence of stress. This may be because of the ability of D-LDH enzyme to use stress produced metabolites for energy production. The oxidation of D-lactate has been coupled to mitochondrial respiratory chain via donating electron to cytochrome c in a variety of organisms including *S. cerevisiae, K. lactis, R. palustris, H. tuberosus and A. thaliana*^13,16,18,28,29,66^. In conditions of stress, MG is overproduced and glyoxalase system is highly active leading to accumulation of D-lactate. Then, D-LDH oxidizes this D-lactate and transfers the electron to respiratory chain, thereby leading to energy production. Thus, D-LDH not only gets rid of the toxic metabolites and their probable harmful consequences on cellular homeostasis, rather employs them to produce energy, ultimately maintaining the cellular growth and yield of plants.

**Figure 9:**
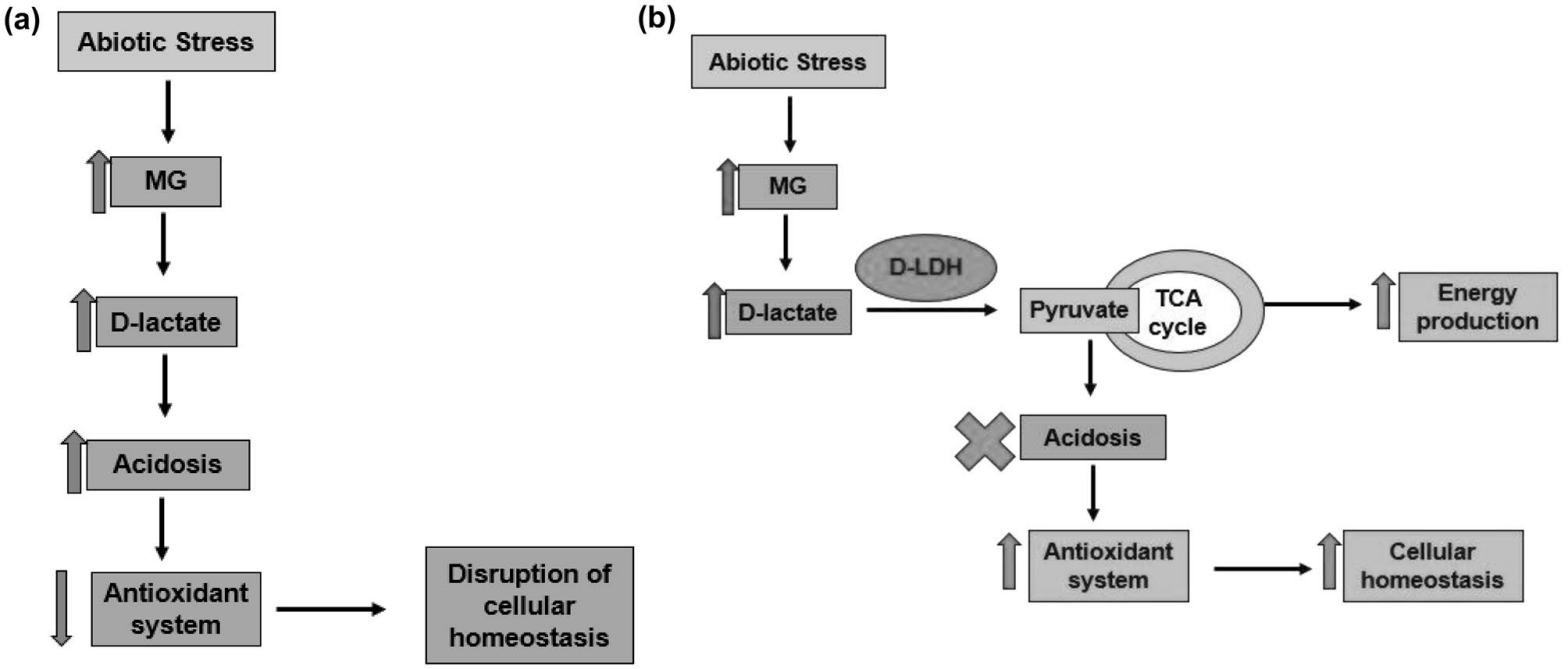
The detoxification mechanism of D-LDH. D-LDH completes the detoxification process of stress induced MG to pyruvate. The figure represents the scenario inside the cell in case of: (**a**) Absence of D-LDH from the cells (*D-LDH *mutants): Abiotic stress leads to an increase in MG levels, leading to a corresponding increase in D-lactate level by the detoxification by glyoxalase enzymes. If, D-LDH is not present, the increased D-lactate accumulates, causing acidosis in the cell. In the prevailing acidic conditions, the antioxidant system is unable to perform actively and ultimately, it leads to spoiling of cellular homeostasis. (**b**) Presence of D-LDH (the transgenic plants): Whereas, if D-LDH is present, it catalyzes D-lactate to pyruvate. Pyruvate performs two important functions, which makes all the difference. Firstly, conversion to pyruvate devoids cell of the toxic acidic conditions, thus, enabling the antioxidant system to maintain cellular homeostasis. Secondly, the formed pyruvate goes to TCA cycle and leads to energy production. Thus, the stress induced toxic metabolite, MG, is employed by D-LDH to provide energy in unfavorable times. In this way, D-LDH overexpressing plants, due to better homeostatic conditions and increased energy production, grow better in abiotic stress conditions.

In case of oxidative stress conditions, D2TG plants possessed more branches and grew bushier. The D2TG plants possess other advantage in addition to increased energy production due to which presence of oxidative stress only contributes in their better growth. In our previous study also, D-LDH emerged as a key player in providing oxidative stress tolerance^31^. D-LDH exerts its antioxidant properties by converting the toxic D-lactate to pyruvate^31^. Pyruvate has multiple antioxidant properties. Firstly, pyruvate can directly and non-enzymatically neutralize peroxides and peroxynitrites. Secondly, pyruvate increases the NADPH/NADP+, the source of reducing power and restores the redox potential of GSH, the major antioxidant by providing NADPH reducing equivalents. This occurs by two different mechanisms. Pyruvate carboxylation by certain enzymes such as pyruvate carboxylase and malic enzyme leads to formation of citrate which moves to cytosol where citrate suppresses the activity of enzyme phosphofructokinase and diverts Glucose-6-phosphate to HMP pathway; thereby increasing NADPH formation. Also, citrate acts as the substrate for the NADP+ dependent enzyme isocitrate dehydrogenase^46,67^. This increased NADPH is the prime requirement of enzymatic antioxidant defense system to function properly. So, in a nutshell, because of antioxidant properties of pyruvate, D-LDH is capable of conferring stress tolerance and this conversion of D-lactate to pyruvate by D-LDH helps the plants to grow better specially in oxidative conditions. Since, OsD-LDH2 overexpressing plants grow exceptionally well in oxidative stress and all stresses invariably lead to oxidative stresses, D-LDH overexpressing plants tend to grow well in multiple stresses. In this way, D-LDH completes the detoxification process of MG to pyruvate and diverts it for energy production. Also, the antioxidant system is able to perform better and maintains cellular homeostasis (Fig. 9). On the other hand, if D-LDH is not there, D-lactate accumulation leads to acidosis in the cell which inhibits the cellular functioning.

One of the biggest challenges for the modern sustainable agricultural development is identification of appropriate candidate genes, engineering which can generate stress tolerant varieties^68,69^. OsD-LDH2, being the most efficient D-LDH enzyme known till date, might be one such candidate conferring tolerance to multiple abiotic stresses. OsD-LDH2, acts as an extension of glyoxalase pathway working on its end product D-lactate, catalyzing it to pyruvate and thus, employing the stress generated methylglyoxal for energy production and helping the plant survive in unfavorable times. This is the first study that correlates D-LDH with abiotic stress tolerance. Previous studies have reported overexpression of both *GLYI* and *GLYII* together provides more stress tolerance than either of the enzymes alone^3,70^. With such an impacting role of *D-LDH* in abiotic stress tolerance, stacking of the three genes of MG detoxification, *GLYI*, *GLYII* and *D-LDH* altogether may provide a new direction in the field of generating stress resilient crop varieties.

## Supplementary information

Supplementary information.

## Data Availability

All data generated or analyzed during this study are included in this published article (and its Supplementary Information files).
