# Supplementary material for: A D-lactate dehydrogenase from rice is involved in conferring tolerance to multiple abiotic stresses by maintaining cellular homeostasis
Source: Sci Rep. 2020 Jul 30;10:12835. doi: 10.1038/s41598-020-69742-0 (PMC7393112; doi:10.1038/s41598-020-69742-0)
Supplement: Supplementary file 1 — Supplementary information. [file 41598_2020_69742_MOESM1_ESM.pdf]

A D-lactate dehydrogenase from rice is involved in conferring tolerance to multiple abiotic stresses by maintaining cellular homeostasis

Muskan Jain, Sakshi Aggarwal, Preeti Nagar, Roopam Tiwari, Ananda Mustafiz*

Laboratory of Plant Molecular Biology, Faculty of Life Sciences and Biotechnology, South Asian University, Akbar Bhawan, Chanakyapuri, New Delhi 110021, India

***Corresponding Author:** Ananda Mustafiz, Laboratory of Plant Molecular Biology, Faculty of Life Sciences and Biotechnology, South Asian University, Akbar Bhawan, Chanakyapuri, New Delhi 110021, India, **Phone:** +91-011-24195286, **Email:** amustafiz@sau.ac.in

Supplementary Figures

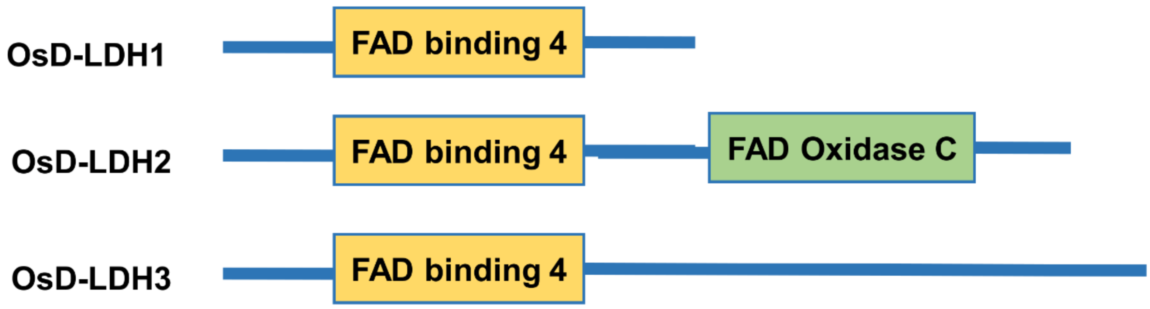

Supplementary Figure S1: Block diagram representing the protein domains. Based on the *in silico* analysis, the three putative D-LDH proteins were identified. All of these contained FAD binding 4 domain, whereas only OsD-LDH2 protein contained another domain FAD oxidase c. OsD-LDH2 was the only putatively active protein identified.

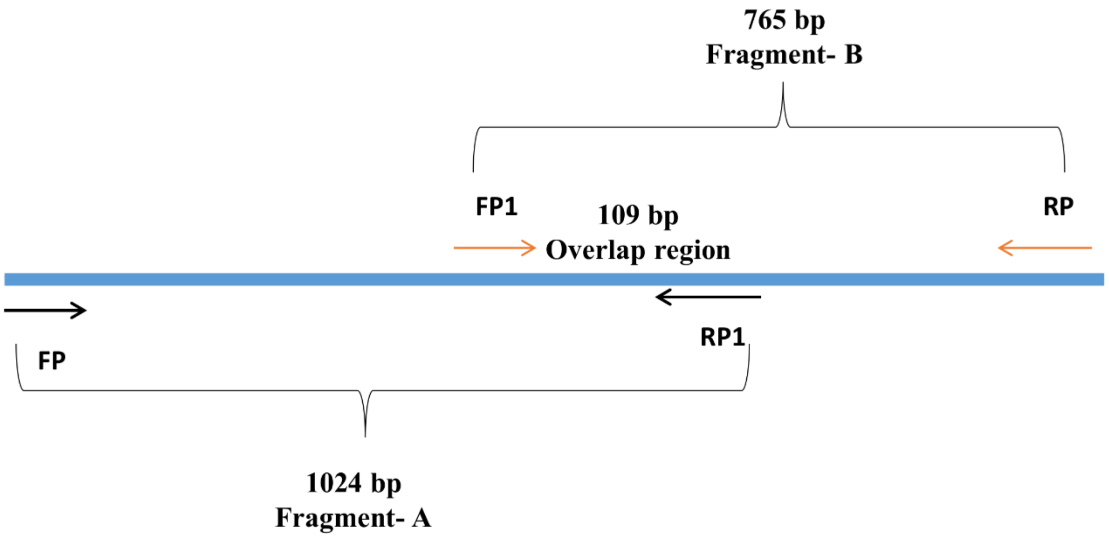

Supplementary Figure S2: A schematic diagram representing the plan of overlapping PCR for amplification of full length gene *OsD-LDH2*. Fragment A and B were amplified separately using primers FP, RP1 and FP1, RP respectively. The primers were designed in such a way that the two fragments had an overlap of 109 bp, using which amplification of full length gene was done.

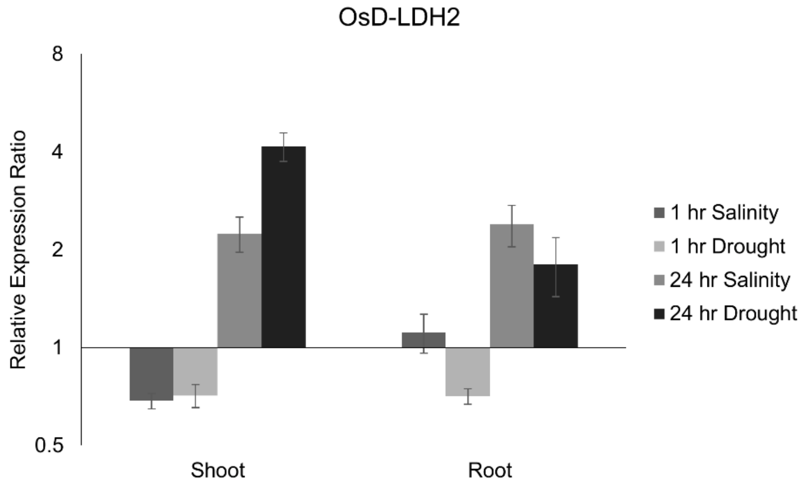

Supplementary Figure S3: Expression profile of *OsD-LDH2* gene in response to different abiotic stress conditions: The expression level of OsD-LDH2 gene in response to different abiotic stress condition [drought and salinity (200mM)] for 1 h and 24 h duration in shoot and root tissue was obtained by qRT-PCR. Expression level of OsD-LDH2 in control sample without any stress was set as 1 and relative expression ratio was plotted in form of bar graph. All qRT-PCR experiments were done with three biological replicates and three technical replicates. The standard deviation of three biological replicates has been shown by error bars.

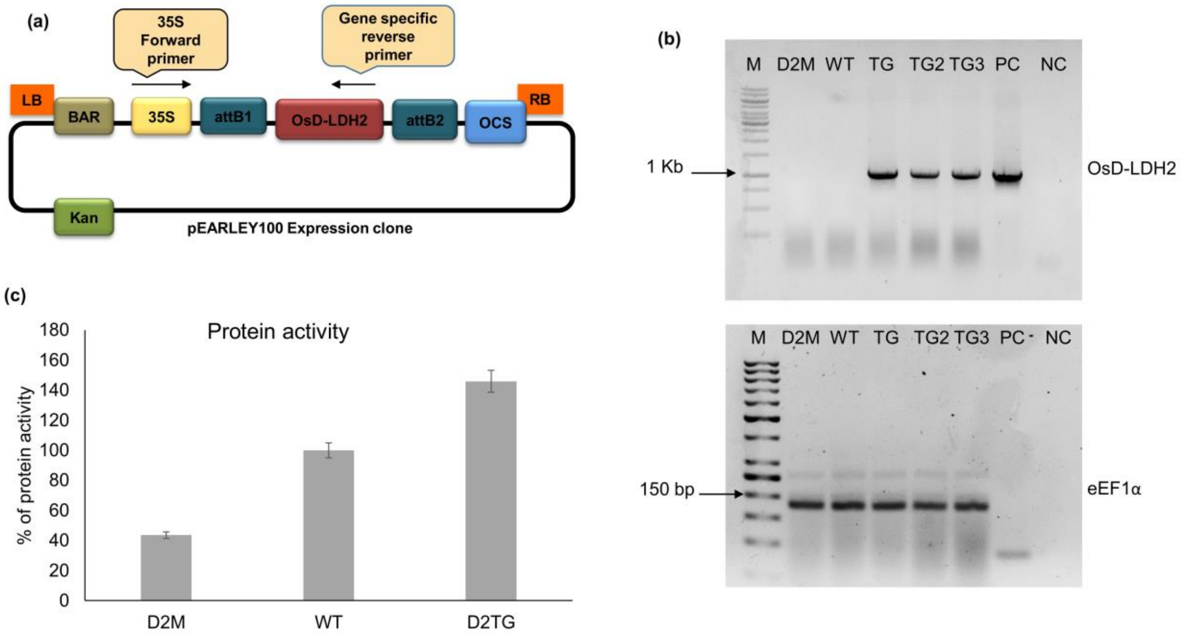

Supplementary Figure S4: Generation and confirmation of *OsD-LDH2* overexpressing transgenic Arabidopsis plants at DNA and protein level. (a) Schematic representation of pEARLEY100+OsD-LDH2 (expression clone) construct used for transformation of *Arabidopsis thaliana* plant via floral-dip. (b) Putative single insertion homozygous plants (T4 generation transgenic plants) were screened by polymerase chain reaction (PCR) using genomic DNA as the template and 35S CaMV promoter specific forward primer (FP) and OsD-

LDH2 gene fragment 1 specific reverse primers (RP). Here, M: D-LDH mutant line, WT: wild type plant, TG1, TG2 and TG3: OsD-LDH2 overexpressing transgenic lines 1, 2 and 3. PC: positive control, NC: negative control. A band around 1100 bp was expected from positive lines. The housekeeping gene eEF1α was also amplified from genomic DNA of all the respective plants and the corresponding bands obtained are shown in the lower panel. A band around 184 bp was expected. For the positive control cDNA was used, which gave a band around 76 bp. (c) Graph showing the D-LDH activity of the protein extract prepared from D-LDH mutant, wild type and OsD-LDH2 overexpressing transgenic tissue. The D-LDH activity assay was done using D-lactate as substrate and measuring the absorbance at 600 nm. M: D-LDH mutant, WT: wild type, D2TG: OsD-LDH2 transgenic line.

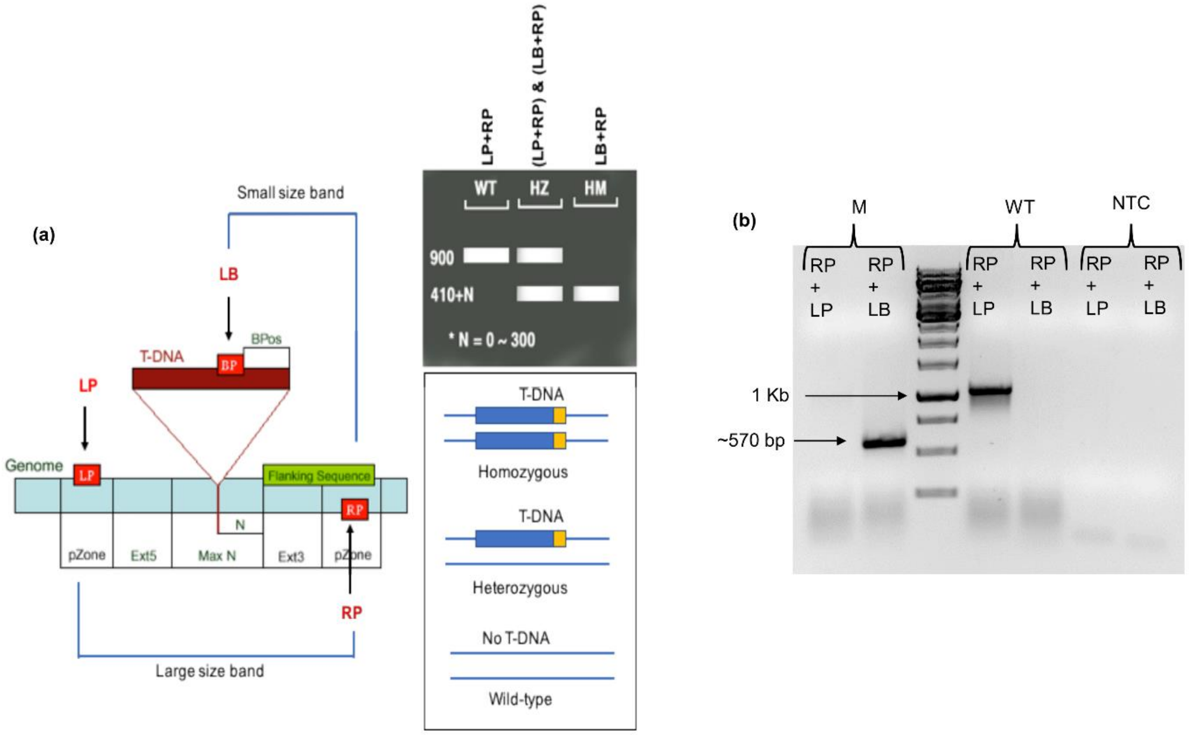

Supplementary Figure S5: Confirmation of the genotype of Arabidopsis mutant line obtained. (a) A schematic diagram showing the expected bands from PCR to confirm the genotype of mutant plant. Using genomic DNA as template, two separate PCR reactions with LP+RP and LB+RP primers were set for each T-DNA insertional mutant line obtained from ABRC. Amplification product from a heterozygous individual will be obtained from both LP+RP and LB+RP primer combinations as it contained single T-DNA insert in either of the alleles. Whereas, amplification product from a homozygous individual will only be obtained from LB+RP primer combination as it had T-DNA inserted in both copies of the gene. On the contrary, wild-type plant gave amplification from LP+RP primers no band from LB+RP primer combination due to absence of T-DNA insert in either of the alleles. Here, LP: Left primer; RP: Right primer and LB: Left border primer. (b) 1% EtBr stained agarose gel showing the PCR amplification product using LP+RP and LB+RP primers from the mutant and wild type genomic DNA. The amplification product from both the reactions was loaded onto 1 % agarose gel to confirm the genotype of plants. The mutant plant gave a single band with LB+RP around 570 bp whereas wild type plant gave band from the other PCR, LP+RP around 1000 bp.

Supplementary Tables

Table S1: List of D-LDH gene family members from rice along with their chromosomal location, nucleotide length, polypeptide length, CDS coordinates and domains present in the different protein members.

Gene	Protein	Chromosome no.	Locus identifier	Total CDS length (bp)	Polypeptide length (aa)	CDS coordinates (5'-3')	Domains
OsD-LDH1	OsD-LDH1	7	Loc_Os07g06890.1	756	252	3370020-3363008	FAD binding 4
OsD-LDH2	OsD-LDH2	7	Loc_Os07g08950.1	1680	560	4652793-4660423	FAD binding 4<br><br>FAD oxidase c
OsD-LDH3.1	OsD-LDH3.1	10	Loc_Os10g25780.1	1770	590	13362322-13359545	FAD binding 4
OsD-LDH3.2	OsD-LDH3.2	10	Loc_Os10g25780.2	1686	562	13363225-13359545	FAD binding 4
OsD-LDH3.3	OsD-LDH3.3	10	Loc_Os10g25780.3	1686	562	13361910-13359545	FAD binding 4

Table S2: A list of primers used in the study.

Primer Name	Sequence
OsD-LDH2 FP	CGGGATCCATGGCGAGGCGCGCGGCGGCGG
OsD-LDH2 RP	CCGCTCGAGCTACAGGACGGACTGTGGGAGA
OsD-LDH2 FP1	CTTACTCCTGCAAAGCTACCATCAAC
OsD-LDH2 RP1	GAATCTCACCCAGGCTCCTCCTGGCTGCCAG
OsD-LDH2 RT FP	TCAAGGTCAGCAACGCTCTT
OsD-LDH2 RT RP	ATTCATGCGTGCGCTTTGTT
OsD-LDH2 GW FP	GGGGACAAGTTTGTACAAAAAAGCAGGCTATGGCGAGGCGCGCGGCGGCGG
OsD-LDH2 GW RP	GGGGACCACTTTGTACAAGAAAGCTGGGTCCTACAGGACGGACTGTGGGAGA
SALK OsD-LDH2 LP	GAGGTGCTGCAAAATTTGAAC
SALK OsD-LDH2 RP	TGTTGGCTAACCTGGATCAAG
LB	CAAGCCTGATTGGGAGAAAA
